# Psychological impact of COVID-19 and contributing factors of students’ preventive behavior based on HBM in Gondar, Ethiopia

**DOI:** 10.1371/journal.pone.0258642

**Published:** 2021-10-25

**Authors:** Ayenew Kassie Tesema, Kegnie Shitu, Asmamaw Adugna, Simegnew Handebo

**Affiliations:** Department of Health Education and Behavioural Sciences, College of Medicine and Health Sciences University of Gondar, Gondar, Ethiopia; Qazvin University of Medical Sciences, ISLAMIC REPUBLIC OF IRAN

## Abstract

**Background:**

The Ethiopian Federal government has locked down schools as one measure to contain Covid-19 pandemic. Psychological effect of COVID-19 on students is increased due to the reopening of schools. The psychological effect of the pandemic is increasing along with physical aspect of health. Therefore, this study aimed to assess the psychological impact of Covid-19 and its contributing factors of students’ behavior in Ethiopia.

**Methods:**

A cross sectional design was conducted from November to December 2020. Data were collected using pre tested self- administered questionnaire from secondary school students in Gondar city North West Ethiopia. Stratified simple random sampling technique was used to select 403 secondary school students. Data were entered and cleaned with Epidata version 4.62 and exported for analysis STATA version 14. Multivariable logistic regression and multiple linear regression were used to show the association of dependent and independent variables. Independent variables in relation to dependent variable measured using odd ratios and B coefficient with 95% confidence interval for Covid-19 anxiety and preventive behavior of Covid-19 respectively were used.

**Results:**

A total of 370 students were participated giving response rate of 92%. The prevalence of Covid-19 anxiety and obsession among secondary school students were 38.1% and 40.27% respectively. Being 11^th^grade 54% (AOR = 0.46; 95%CI:0.22, 0.95) and increased knowledge16% (AOR = 0.84;95%CI: 0.77, 0.89) score associated with decreased COVID-19 anxiety while Covid-19 obsession, 14.51 times (AOR = 14.51;95%CI: 8.05, 26.17), and being female 1.6 times (AOR = 1.6; 95%CI: 1.01, 2.51) increased Covid-19 Anxiety. Furthermore, increased self-efficacy 0.5 times (B = 0.5; 95%CI: 0.28, 0.62), and increased cues to action 0.4 times (B = 0.4; 95% CI: 0.19, 0.69) increased preventive behavior of Covid-19 while perceived barrier 0.1 times (B = -0.1; 95%CI:-0.22, 0.01) decrease preventive behavior of Covid-19.

**Conclusion:**

Almost two individuals of five participants developed COVID-19 anxiety and COVID-19 obsession. Being grade 11^th^ and knowledge were negatively associated with anxiety while being female and being obsessed with COVID-19 were positively associated with anxiety. No variable was associated with obsession of Covid-19. Intervention is needed to reduce anxiety among females. Furthermore, perceived barrier, self-efficacy and cues to action were significant factors of preventive behaviour of Covid-19. Therefore, to increase preventive behaviour of Covid-19, information, education and communication and behavioural change communication should be targeted on reducing barriers and increasing motivations and confidences.

## Background

Coronavirus disease 2019 (COVID-19) is a viral infection that causes serious respiratory illness such as pneumonia and lung failure and it was first reported in Wuhan, the capital of Hubei, China [1]. Even though the clinical symptoms of COVID-19 are indicated nonspecific, the commonly reported symptoms include fever, cough, myalgia, and fatigue. In addition to this patient may initially present with diarrhea nausea, and headache usually a few days before developing a fever [2]. According to the European Commission report of February 9, 2021, over 107 million cases and 2.33 million deaths have been reported worldwide. According to the report Ethiopia is one of the African countries which have reported the most deaths in Africa, where more than 142,994 cases and 2,156 individuals were died until February 9, 2021 [3]. As day to day reports have shown the pandemic is increasing in Ethiopia from time to time [4].

The Covid-19 has had numerous effect on different occupations and create psychological impact among individuals [5]. A systematic indicated revealed that Covid-19 fear and anxiety contributed on job insecurity and turn over [6]. Thus, schools are institutions where students are gathered together to learn, which makes physical distancing difficult and in turn impose increased risk of the transmission of the pandemic from on to other unless adequate precaution measures are taken by the school and the students too [7]. Following the introduction of the pandemic, the Ethiopian Federal government has locked down schools as one measure to contain the pandemic. However, after eight months of lockdown the government declare school re-opening in the country. In this point of view student’s compliance to the preventive behaviors for the prevention of the pandemic is mandatory to protect themselves from contracting the infection in addition to the school’s readiness to combat the pandemic [8, 9].

Anxiety is a self-reaction or filling worry to a noncommittal or unknown threat. This manifest it-self when the person believes that dangerous could take place on everyone’s body [10]. Studies on Anxiety and stress suggested that the magnitude of anxiety among general population was 31.9% [11] and a study among Bangladesh university students revealed that the magnitude of sever and moderate anxiety were 18.1%, 15% respectively. Evidences suggested that individuals are experiencing high level of anxiety during COVID-19 [10–12].The psychological impact like anxiety, obsession and fear of Covid-19 varies across countries. For example, in India it ranges from 20%to 25% [13, 14] a systematic review among health workers revealed that the pooled prevalence was 37% [5] Studies examined that sociodemographic the factors such as lower age, female sex, household income, and social support were the determinant factors of Anxiety and obsession [13, 15, 16]. Therefore, understanding and identifying the psychological impact and the contributing factors is vital to enhance preventive behavior of Covid-19. And the application of theories of health promotion help to understand the determinant factors of Covid-19 preventive behavior.

Health belief model is one of the most effective theory for identifying factors affecting on the perception of the behavior of interest [14–18]. The model suggests that changes in preventive health behavior are originally based on six constructs; perceived susceptibility: perceived severity, perceived benefits, perceived barriers, self-efficacy and cues to action [19–21]. The Health belief model (HBM) constructs are factors determine Covid-19 preventive behavior. Studies demonstrated that perceived susceptibility, perceived benefit and self-efficacy increase Covid-19 preventive behavior [22, 23] while perceived barrier decrease preventive behavior [21, 22]. The aim of this study was to assess psychological impact of COVID-19 and its determinants among secondary school students in Gondar city North West Ethiopia.

## Methods

### Study design and setting

An institution based cross-sectional study was conducted from November- December, 2020. The study was conducted in Gondar city administration. Gondar city is located at about 727 km away from Addis Ababa, the capital city of Ethiopia, and 180 km away from Bahir Dar the capital city of Amhara Regional State. Gondar city has the total area of 192.3 Sq.KM with a total population of 338, 646. The town is composed of 6 sub cities. There are 51 private and public health facility in the city administration. Of which 43 health institutions are private, with one private general hospital and one public comprehensive specialized referral teaching hospital. In addition to this there are 8 dental clinics in the city. In the city there are 17 Government and Private secondary schools (Grade 9 to Grade 12) with more than 23,200 students among which 12,225 are young female students [24].

### Study population

All students who attended in the selected secondary schools in Gondar city were included in to the study and students who were out of school during the data collection period were excluded from the study.

### Sample size determination and sampling procedure

Sample size was determined by a single population proportion formula which is used in previous research. The total sample used was 403.

Stratified simple random sampling technique was used to select the study participants. First, stratification was done based on school ownership into private and governmental schools. Then, four governmental and two private secondary schools were selected on random basis. Finally, study participants were selected randomly based on their class roaster using Microsoft excel random number generator.

### Study variables

The outcome variable was COVID-19 Anxiety and obsession of Covid-19. The independent variables were Sociodemographic factors (age, sex, educational level, religion, maternal occupation, paternal occupation, maternal educational status, paternal educational status, marital status), social support, knowledge of COVID-19, health belief model variables (perceived severity, Perceived susceptibility, perceived barriers, Perceived benefits and Cues to action).

### Measurements

#### Coronavirus anxiety scale

It is obvious that treatment of Covid-19 gives little attention to the fear of COVID-19. It is due to the lack of an appropriate psychometric instrument. So using valid instrument to determine the participant’s anxiety of COVID-19 is important for health practitioners and designers. The CAS has five items and is rated on a 5-point scale from 0 (not at all) to 4 (nearly every day over the last two weeks). A total score of ≥5 was reported as anxious [25]. The reliability of the items with Cronbach’s was α = 81.6%.

#### Obsession with Covid-19scale

The Obsession with COVID-19 Scale (OCS) is a self-report type mental health screener that measures persistent disturbing thinking associated with COVID-19. The OCS has four items each rated on a 5-point scale from 0 (not at all) to 4 (nearly every day over the last two weeks). A total score ≥7 indicates probable dysfunctional thinking about COVID-19 [19, 26]. The reliability of the items with Cronbach’s was α = 81.4%.

#### Perceived susceptibility

It is one’s perception of the risk to contract COVID-19 and it was measured by six items having a five-point Likert scale. Its score ranged from 6–30. The higher score indicates higher perceived susceptibility towards COVID-19 [12] and the internal consistency was (Cronbach’s α = 75.3%).

#### Perceived severity

It is one’s perception of the seriousness of COVID-19 and it was measured by 5 items having five-point Likert scale. Its score ranged from 5–25. The higher score indicates higher perceived susceptibility towards COVID-19 [12] and the internal consistency was (Cronbach’s α = 76.2%).

#### Perceived benefit

It is one’s perception of the benefits of wearing a facemask, keeping physical distance, and washing hands frequently for the prevention of COVID-19 and it was measured by five items having a five-point Likert scale. Its score ranged from 5–25. The higher score indicates higher perceived benefits of performing recommended preventive behaviors of COVID-19 [27] and with high internal consistency (Cronbach’s was α = 80.3%).

#### Perceived barriers

It is one’s perception of the factors that restrict an individual to do COVID-19 preventive measures and it was measured by seven items having a five-point Likert scale. The higher score indicates higher perceived barriers to avoid behavioral risk behaviors of COVID-19 [28] and the internal consistency was (Cronbach’s α = 75.9%).

#### Self-efficacy

It is one’s confidence to execute recommended preventive measures of COVID-19 and it was measured by four items having a five-point Likert scale. Its score ranged from 4 to 20. A higher score indicates one’s higher self-efficacy/confidence to execute the recommended measures [22, 23] with high internal consistency (Cronbach’s α = 78.6%.

#### Cues to action

It refers to the impact of triggering Media, bodily testimonials on once compliance behavior to the preventive measures of COVID-19. It was measured by three items having five-point Likert scale. Its score ranged from 3 to 15. The higher score indicates the impact of cues to execute preventive behaviors [28] with high internal consistency (Cronbach’s α = 76.1%).

#### Preventive health behaviors

Refers to the participant’s practice concerning, hand washing, physical distancing, and face mask-wearing, to prevent COVID-19 infection. It was measured by eight items having a five-point response rate ranging from 1 (Never) to 5 (always). The composite score of the preventive behaviors ranged from 8 to 40. A higher score indicates compliance behavior [19, 23, 25, 29]. The internal consistency reliability with Cronbach alpha was76.6%.

#### Knowledge of COVID-19

Refers to participant’s cognition of symptoms, nature, and preventive measures of COVID-19. It was measured by 17 items having three response categories (1 = True, 2 = False, and 3 = I don’t know). Response categories merged into 1 for correct responses and 0 for incorrect responses. The composite score ranged from 0–17 and the higher score indicates better knowledge of the participant about COVID-19 [30]. The internal consistency reliability with Cronbach’s’ alpha was 81.6%.

#### Social support

It is defined as self-reported supports that participants receive from others. It was measured using the Oslo social support scale (OSSS-3) containing three items and the sum score ranging from 3 to 14, a score of 3 to 8 categorized as poor social support, a score of 9 to 11 categorized as moderate, and a score of 12 to 14 categorized as strong social support [29, 31].

### Data collection tools and procedures

Data was collected by a pretested self-administered structured questionnaire. The questionnaire was prepared by the investigator after reviewing different pieces literatures [11, 12, 15, 26–28, 32–35]. The questionnaire contained six sections. The sections were sociodemographic characteristics of participants, the social support related questions, the health belief model questions, and preventive health behavior of COVID-19 questions, knowledge questions and the last section was about Covid-19 anxiety and obsession questions.

### Data quality assurance

Initially, the instrument (questionnaire) was developed in English by the investigators then forward and backward translation was done by both Amharic and English versed individuals to keep its consistency. Necessary amendments were made on the instrument based on translation reports from the translators. A one-day training was given to both the data collectors and supervisors by the principal investigators about the purpose of the study, data collection procedures and ethical issues during data collection.

We conduct content validity test by participating 5 experts from health behavior, infectious disease, and COVID-19 pandemic response team. It was determined by Item level Content Validity Index (I-CVI) of 0.78 or higher, Scale level Content Validity Index by Universal Agreement (S- CVI/UA) 0f 0.8 or higher and Scale level Content Validity Index by Average (S-CVI/Ave) 0.9 or higher. Moreover, a pretest was done at secondary school which were not selected for the main study among with a 5% of the total sample size for the assessment of the questionnaire clarity, and sociocultural compatibility. In addition to this, the reliability of the instrument was checked.

### Data analysis

Data were cleaned and coded data with EPI DATA version 4.6.2 and exported to STATA version 14 for analysis Descriptive statistics such as means standard deviations, frequencies, and proportions was computed. A Confidence level of 95% and p-value of less than 0.05 were used to determine statistical significance. Binary logistic regression employed to identify factors associated with Covid-19 Anxiety. Those variables with p-value less than or equal to 0.2 from bi-variable analysis were candidates for multivariable analysis. In addition multiple linear regression was done to identify factors associated with preventive behavior of Covid-19. The assumptions were assessed before employing regression. Multivariable analysis was conducted to control potential confounders and to declare the significant of the association, p-value < 0.05 used. Moreover, the magnitude of the association between different independent variables in relation to dependent variable measured using odd ratios with 95% confidence interval. Indeed, the Hosmer-Lemshow goodness of fit test used to test the model fitness.

### Ethical approval and consent to participation

Ethical clearance obtained from the Institutional Review Board of University of Gondar with ID number V/P/RCS/05/588/2020. Letter of permission obtained from Gondar city education office. After the purpose and objective of the study have been informed, written consent obtained from each study participants. For those participants whose less than 18 years old consent was obtain from their parents or guardian. All participants also informed that participation was on voluntary basis and they can withdraw from the study at any time if they were not comfortable about the questionnaire. In order to keep confidentiality of any information provided by study subjects, the data collection procedure was kept anonymously.

## Results

### Sociodemographic characteristics

A total of 370 students were participated giving a response rate of 92%. About 59.73% of participants age were eighteen and above. More than half (51.89%) were females and 84.59% were unmarried. Most of them (87.57%) were orthodox Christian religion followers and about 42.43% of participants were grade 10 students. The majority (70.81%) of participant’s mothers were housewives in occupations (Table 1).

**Table 1:** Sociodemographic characteristics of Gondar city secondary school students, North West Ethiopia, 2021(n = 370).

Variable	Category	Frequency	Percent
Age	<18years	149	40.27
	> = 18years	221	59.73
Sex	Male	178	48.11
	Female	192	51.89
Grade	10^th^	157	42.43
	11^th^	93	25.14
	12^th^	120	32.43
Marital status	Unmarried	313	84.59
	Married	57	15.41
Religion	Orthodox Christian	324	87.57
	Muslim	39	10.54
	Others	7	1.89
Maternal occupation	Housewife	262	70.81
	Employee	60	16.22
	Marchant	33	8.92
	Others	15	4.05
Paternal occupation	Employee	134	36.22
	Farmer	121	32.70
	Marchant	93	25.14
	Others	22	5.95
Maternal educational status	Unable to read and write	131	35.41
	Able to read and write	89	24.05
	Primary	59	15.95
	Secondary and above	91	24.59
Paternal educational status	Unable to read and write	64	17.30
	Able to read and write	116	31.35
	Primary	59	15.95
	Secondary and above	131	35.41

#### Social support

More than one-fourth of participants had 1 to 2 close people who can support during great personal problems. About 41.08% of participants reported that it is easy to get particular help from neighbors if they need it while 38.38% perceived that people show a lot of interest and concern in what they do. About 45.68% of them had medium social support as shown in (Table 2).

**Table 2:** Social support of Gondar city secondary school students, North West Ethiopia, 202 (n = 370).

Variables	Category	Frequency	Percent
How many people are so close to you that you can count on them if you have great personal problems?	None	35	9.46
	1–2	95	25.68
	3–5	64	17.30
	+5	176	47.57
How much interest and concern do people show in what you do?	None	9	2.43
	Little	14	3.78
	Uncertain	98	26.49
	Some	107	28.92
	A lot	142	38.38
How easy is it to get practical help from neighbors if you should need it?	Very difficult	36	9.73
	Difficult	35	9.46
	Possible	152	41.08
	Easy	67	18.11
	Very easy	80	21.62
Social support level	Poor	76	20.54
	Medium	169	45.68
	Strong	125	33.78

### COVID-19 perception, knowledge and preventive behaviour

The perception of participants was assessed using eight health belief model constructs; perceived susceptibility, perceived severity, perceived benefit, perceived barrier, self-efficacy, and cues to action. To show the descriptive statistics, the median and interquartile range was used because the data distribution of all the variables were not normally distributed. The median score of perceived susceptibility and perceived severity were 17 and16 respectively (S1 Table). The median knowledge and preventable health behavior of COVID-19 were 13 and 23 respectively (Table 3).

**Table 3:** COVID-19 perception, knowledge and preventive behaviour of Gondar city secondary school students, North West Ethiopia, 2021(n = 370).

Variable	Minimum	Maximum	Midian	Interquartile range
Perceived susceptibility	6	30	17	6
Perceived severity	5	25	16	7
Perceived benefit	5	25	20	6
Perceived barrier	7	34	20	8
Self-efficacy	4	20	12	7
Cues to action	3	15	12	4
Knowledge	0	17	13	4
Preventive health behaviour	8	40	23	9

### Anxiety and obsession of COVID-19 among students

**Fig 1:**
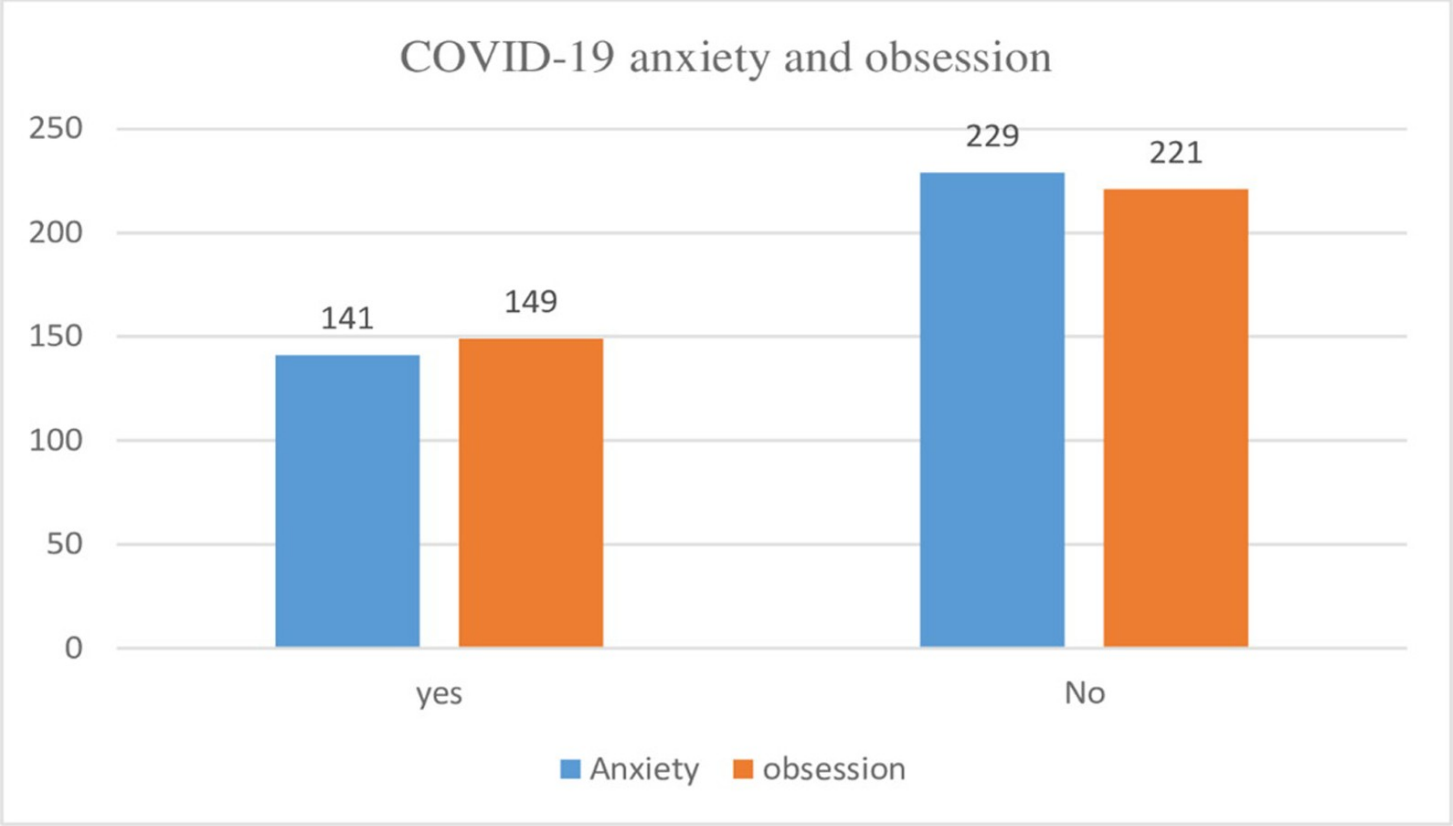
Percentage of Covid-19 anxiety and obsession of Covid-19 among Gondar city secondary school students, North West Ethiopia, 2021(n = 370).

The prevalence of anxiety and obsession among secondary school students were 38.1% (95%CI; 33.3% -43.2%;) and 40.27% (95% CI;.35.4%- 45.4%) (Fig 1).

### Contributing factors of COVID-19 anxiety and obsession

Bivariable and multivariable logistic regression was conducted to identify factors that determine COVID-19 anxiety and obsession of participants. Two models were fitted independently for Anxiety and obsession each. But no factor was associated with Covid-19 obsession. On Bivariable regression female sex, being grade 11th students, maternal occupation, COVID-19 obsession, prevention behavior, and knowledge were competent variables for multivariable logistic regression of Covid-19 Anxiety. Of those being grade 11th, COVID-19 obsession, female sex, and knowledge were statistically significant variables.

Being female sex, and Covid-19 preventive behavior had direct association with anxiety. Being female increased the odds anxiety by 1.6 times (AOR = 1.6; 95%CI: 1.01, 2.51) than males. The odds of anxiety among individuals with COVID-19 obsession were 14.51 (AOR = 14.51:95%CI; 8.05, 26.17) times more likely to be anxious than individuals with no COVID-19 obsession. Knowledge towards COVID-19 and participants’ educational level had an inverse relationship with Covid-19 anxiety. Keeping other variables constant the odds of COVID-19 anxiety decreased by 16% (AOR = 0.84:95%CI; 0.77, 0.89) as a unit knowledge score increased and the odds of anxiety decreased by 54% (AOR = 0.46; 95%CI 0.22, 0.95) among grade 11th students than grade 10 students (Table 4).

**Table 4:** Multivariable logistic regression of anxiety and its determinant factors of Gondar city secondary school students.

variable		Covid-19 related Anxiety		AOR (95%CI)	p-value
		Yes (%)	No(%)		
Sex	Male	59 (33.15)	119(66.85)	1	
	Female	42.71(42.71)	110(57.29)	1.6(1.01,2.51)*	0.04
Grade of students	Grade 10^th^	67 (42.68)	90(57.32)	1	
	Grade 11^th^	29(31.18)	64(68.82)	0.46(0.22,0.95) *	0.04
	Grade 12^th^	45(37.50)	75(62.50)	0.56(0.29,1.07)	0.08
Maternal occupation	Housewife	107(40.84)	155(59.16)	1	
	Employee	18(30.00)	42(70.00)	0.65(0.29,1.45)	0.30
	Marchant	11(33.33)	22(66.67)	0.59(0.21,1.67)	0.32
	Others	5(33.33)	10(66.67)	0.76(0.18,3.19)	0.72
Obsession of COVID-19	Yes	106(71.14)	43(28.86)	14.51(8.05, 26.17) *	<0.001
	No	35(15.84)	186(84.16)	1	
knowledge				0.84(0.77, 0.91)*	<0.001

Note * statistically significant variables at p<0.05.

### Contributing factors of COVID-19 preventive behaviour based on health belief model

At the very beginning, simple linear regression was conducted, then those variables with a p value of <0.2 were candidates for multivariable linear regression. The health belief model constructs were the only determinants of Covid-19 preventive behavior. Perceived barrier, cues to action, and self-efficacy were statistically significant variables having association with Covid-19 preventive behavior. Self-efficacy was the strongest (β = 0.29) determinant factor followed by cues to action (β = 0.21). Furthermore, cues to action and self-efficacy associated positively while perceived barrier associated negatively. The Covid-19 preventive behavior increased by 0.4 (B = 0.4; 95%CI: 0.19, 0.69) as a unit score increase in cues to action keeping other variables constant. Keeping other variables constant as a unit score increase in self-efficacy the Covid-19 preventive behavior increased by 0.5(B = 0.5;95%CI:0.28,0.62). Perceived barrier was the other construct of health belief model had negative association with preventive behavior. As a unit score increase in perceived barrier Covid-19 preventive behavior increased by 0.1(B = -0.1;95%CI:-0.22,0.01) given that other variables are kept constant (Table 5).

**Table 5:** Multivariable linear regression of COVID-19 prevention based HBM and its determinant factors of Gondar city Secondary school students.

		Simple linear regression	Multiple linear regression		
variable		B(95% CI)	Standardized β	B (95%CI)	p-value
Educational status	Grade 10^th^			1	1
	Grade 11^th^	-1.2(-2.75, 0.41)	-0.04	-0.7(-2.07,0.76)	0.36
	Grade 12^th^	0.7(-.94, 1.99)	0.04	0.6(-0.73,1.89)	0.38
Social support	Poor			1	1
	Medium	1.1(-.56,2.78)	0.04	0.5(-0.93,2.07)	0.45
	Strong	1.1(-0.66, 2.86)	0.03	0.4(-1.16,1.99)	0.62
Perceived susceptibility		0.1(-0.07,0.21)	-0.04	-0.1 (-0.19,0.07)	0.39
Perceived severity		0.3(0.13,0.4)	0.07	0.1(-.040,0.26)	0.15
Perceived benefit		0.3(0.18, 0.45)	0.02	0.1(-0.12,0.19)	0.64
Perceived barrier		-0.2(-0.29, -0.06)	-0.09	-0.1(-0.22,0.01)*	0.04
Cues to action		0.8(0.63,1.04)	0.21	0.4 (0.19,0.69)*	0.01
Self-Efficacy		0.7(0.54, 0.83)	0.29	0.5(0.28,0.62)*	<0.01
knowledge		0.1(-0.10,0.25)	-0.03	-0.1(-0.22,0.11)	0.53

*Statistically significant at p-value <0.05

## Discussion

The study aimed to assess the psychological impact of COVID-19 and contributing factors of students’ preventive behavior based on Health belief model in Gondar, Ethiopia. Being female, being grade 11^th^, being obsessed and knowledge were associated factors with Covid-19 anxiety while perceived barrier, self- efficacy and cues to action were factors associated Covid-19 preventive behaviour.

The current study revealed that the proportion of COVID-19 anxiety among Gondar city secondary school students was 38.1%. This finding is in line with a study done in Philippines nurses (37.8%) but lower than studies conducted in Bangladesh (87.7%), united Arab Emirates (46.9%), France(60.2%, and Iran(50.9%) [15, 36, 37]. However, this study is higher than a study done in India (25%) [13]. The variation could be difference in the study population and use of different measuring tool. In this study COVID-19 anxiety scale was used but the above mentioned studies except Philippines study used generalised anxiety disorder-7 tool. In addition, in the current study the prevalence of obsession of Covid-19 was 42.27%. This finding is higher than a study done in India [14]. The variation might be due to difference in demography such as educational status. The majority of the educational status of that study were graduates and post graduates and psychological impact is inversely related with educational level. This indicate that interventions and adaptive mechanisms is important for females.

The current study revealed that the sex of was the significant factor of Covid-19 Anxiety. Females were more likely to be anxious than males which is consistent with previous researches [16, 19, 38]. This might be due to influence of biological, cultural and environmental factors. In addition, women expose to more intense perturbation of gonadal steroid and glucocorticoid responsive brain systems. It is important to note that there are large individual differences in the activation effects of reproductive hormones on behavior [39, 40] and a research indicates that women experience typical levels of reproductive hormone changes, but a sub-optimal central nervous system response that leads to negative affect and maladaptive behavior [38, 41].

This study found that the grade level of students associated with a decrease in COVID-19 anxiety. Participants with grade 11^th^ were associated with less likely COVID-19 anxiety. This finding is inconsistent with a study done in Iran which showed that level of anxiety increase as the educational status increase [42]. The current study revealed that anxiety decrease as the COVID-19 knowledge of student’s increases. This finding is inconsistent with studies done in United Arab Emirates, Yemen, and Pakistan [15, 27, 43]. The level of anxiety increase with knowledge while a study was done in Turkey revealed that there is no association between anxiety and knowledge [36]. This may be justified as our study population with increased knowledge may take COVID-19 prevention measures so that they might not develop anxiety.

The finding of this study revealed that COVID-19 obsession was statistically associated with anxiety, individuals with obsession were more likely to develop anxiety. It is supported by study done in India [44]. Individuals who are obsessed are more likely to develop anxiety [45, 46].

It was further found that three constructs of health belief model were significantly associated with Covid-19 preventive behaviour. Perceived barrier inversely related with preventive behaviour of Covid-19 which is supported by a study done in Northern Iran [21]. So that the effect of preventive behaviour of Covid-19 achieved by reducing perceived barriers. It indicates that perceived barrier is an important construct of health belief model even though it had small influence compared to other constructs [47]. Therefore, individuals should overcome the barriers to prevent Covid-19. The other health belief model construct that had direct association with preventive behaviour was self-efficacy which is consistent with previous researches done in Iran and Ethiopia [23, 25]. This implies that those individuals with high confidence and trust to prevent Covid-19 and overcome the barriers to prevent Covid-19 were more likely to prevent the pandemic. Therefore, developing messages that increase self-efficacy in important. It is also the strongest construct of Health belief model. In addition, Those individuals who had high self-efficacy towards the prevention of COVID-19 and had lower perceived barriers as well, they would take COVID-19 preventive measures [18].

The current research revealed that cues to action was one of the factor which affected the preventive behaviour of Covid-19. According to health belief model definition, cues to action is the readiness or motivation of individuals to prevent the pandemic with media, bodily symptoms and testimonies. Therefore, designing messages, testimonies, advertising with media is crucial to prevent Covid-19.

### Limitation of the study

Despite the strengths like showing the psychological impact in the study are, use of new validated Covid-19 and obsession tool for the pandemic, it has some limitations. The first, we couldn’t show the contributing factors of covid-19 obsession because there was no significant variable. The second, it is expected to employee hierarchical or Structural equation model for preventive behaviour of Covid-19 using HBM but the assumptions not fulfilled so forced to conduct linear regression.

## Conclusion

Almost two individuals of five participants developed COVID-19 anxiety and COVID-19 obsession. Being grade 11^th^ and knowledge were negatively associated with anxiety while being female, and being obsessed were positively associated with anxiety. No variable was associated obsession covid-19. Furthermore, perceived barrier, self-efficacy and cues to action were associated with preventive behavior of Covid-19. Therefore, information, education, and communication (IEC) and Behavioural change communication should target on females. Developing tailored messages focusing on reducing barriers, increase testimonies and increase self-confidence is important.

## Supporting information

S1 Table (DOCX)

## Abbreviations

AOR: adjusted odds ratio
BCC: behavioural change communication
CAS: Covid-19 Anxiety Scale
CI: confidence interval
IEC: information education and communication
OCS: obsession corona scale
WHO: World Health Organization
