# Supplementary material for: Psychological impact of COVID-19 and contributing factors of students’ preventive behavior based on HBM in Gondar, Ethiopia
Source: PLoS One. 2021 Oct 25;16(10):e0258642. doi: 10.1371/journal.pone.0258642 (PMC8544846; doi:10.1371/journal.pone.0258642)
Supplement: S1 Table — (DOCX) [file pone.0258642.s001.docx]

S1 Table.

| Constructs | Items | Strongly disagree | Disagree | Neutral | Agree | Strongly agree |
| --- | --- | --- | --- | --- | --- | --- |
| Perceived susceptibility | I consider myself to  be at risk of coronavirus | 61(16.49%) | 128  (34.59%) | 36 (9.73%) | 89 (24.05%) | 56 (15.14%) |
|  | I don't care about this  disease and  do my daily  activities  like before | 64(17.3%) | 85 (22.97%) | 44 (11.89%) | 116 (31.35%) | 61 (16.49%) |
|  | The transmission  power of this  disease is high | 45(12.16%) | 85(22.97%) | 35 (9.46%) | 133 (35.95%) | 72 (19.46%) |
|  | My working condition makes it  more likely  that I will get prone to COVID-19 | 57 (15.41%) | 96(25.95%) | 21 (5.68%) | 111 (30%) | 85 (22.97%) |
|  | My living condition makes it more likely that I will get prone to COVID-19 | 86 (23.24%) | 164 (44.32%) | 34 (9.19%) | 57 (15.41%) | 29 (7.84%) |
| Perceived severity | The thought of being infected by COVID-19 scares me | 60 (16.22%) | 102 (27.57%) | 28 (7.57%) | 112 (30.27%) | 68 (18.38%) |
|  | Having COVID-19  infection is a  hopeless condition | 62 (16.76%) | 147 (39.7% | 37 (10.00%) | 73 (19.73%) | 51 (13.78%) |
|  | COVID-19 infection can lead to death | 29 (7.84%) | 59 (15.95%) | 23 (6.22%) | 168 (45.41%) | 91 (24.59%) |
|  | I am afraid to  even think about the COVID_19 infection | 57 (15.41%) | 124 (33.51%) | 28 (7.57%) | 103 (27.84%) | 58 (15.68%) |
|  | If I had COVID-19  infection, it would be more serious  than any other | 39 (10.54%) | 75 (20.27%) | 26 (7.0%) | 157 (42.43%) | 73 (19.73%) |
| Perceived benefit | Keeping social  distancing at least  for 1 meter  can help me  to prevent  COVI | 31 (8.38%) | 33 (8.92%) | 13 (3.51%) | 164 (44.32%) | 129 (34.86%) |
|  | Frequent hand  washing for at least 20 seconds can  save me from  COVID-19 | 26 (7.03%) | 46 (12.43%) | 13 (3.51%) | 161 (43.51%) | 124 (33.5%) |
|  | Wearing face mask  when I go out can  prevent me from  COVID-19  infection | 19 (5.14%) | 38 (10.27%) | 28 (7.57%) | 153 (41.35%) | 132 (35.68%) |
|  | Avoiding touching eye, face and nose  can benefit me to  prevent COVID-19 | 19 (5.14%) | 54 (14.59%) | 16 (4.32%) | 144 38.92%) | 137 (37.03%) |
|  | I do preventive behaviours daily | 28 (7.57%) | 69 (18.6%) | 30 (8.11%) | 155 (41.89%) | 88 (23.78%) |
| Perceived barrier | It is difficult to wash  Hands regularly with soap and water | 85 (22.97%) | 159 (42.97%) | 25 (6.76%) | 63 (17.03%) | 38 (10.27%) |
|  | For me it is difficult to buy alcohol | 55 (14.86%) | 150 (40.54%) | 30 (8.1%) | 95 (25.68%) | 40 (10.81%) |
|  | My poor economic  status will preclude me from doing  recommended preventive measures | 54 (14.59%) | 116 (31.35%) | 42 (11.35%) | 104 (28.11%) | 54 (14.59%) |
|  | It is difficult not to touch hands, mouth, nose and eyes | 42 (11.35%) | 82 (22.16%) | 23 (6.22%) | 135 (36.49%) | 88 (23.78%) |
|  | For me, staying at home to prevent the  disease is difficult | 49 (13.24%) | 97 (26.22%) | 26 (7.0%) | 120 (32.43%) | 78 (21.08%) |
|  | I have limited  knowledge about  recommended  preventive health  behaviours | 74 (20.00%) | 161 (43.51%) | 32 (8.65%) | 69 (18.6%) | 34 (9.19%) |
|  | Doing preventive behaviors daily will interfere with my routine activities | 67 (18.11%) | 154 (41.62%) | 35 (9.46%) | 72 (19.46%) | 42 (11.35%) |
| Self -efficacy | I am confident that I can wash my hand | 37 (10.0%) | 121 (32.70%) | 46 (12.43%) | 114 (30.81%) | 52 (14.05%) |
|  | I am sure that I can keep 2 meter distance |  |  |  |  |  |
|  | I am confident to wear mask when going out home | 39 (10.54%) | 120 (32.43%) | 54 (14.59%) | 116 (31.35%) | 41 (11.08%) |
|  | I am confident  that I can avoid  touching face | 56 (15.14%) | 134 (36.22%) | 46 (12.43%) | 98 (26.49%) | 36 (9.73%) |
| Cues to action | Watching people like me die because of COVID-19  makes me to do  preventive | 27 (7.30%) | 58 (15.68%) | 45 (12.16%) | 158 (42.70%) | 82 (22.16%) |
|  | Posters, and public  Health communication on messages help me to engage in prevent | 22 (5.95%) | 61 (16.49%) | 54 (14.59%) | 175 (47.30%) | 58 (15.68%) |
|  | Watching people like me die because of COVID-19 makes me to do prevent | 22 (5.95%) | 48 (12.97%) | 46 (12.43%) | 164 (44.32%) | 90 (24.32%) |

<https://www.kaggle.com/ayenewkassie/psychological-impact-of-covid19>
